# Patient-perceived factors on treatment satisfaction in early onset scoliosis treated surgically with a minimum of ten years

**DOI:** 10.1186/s13018-024-04993-5

**Published:** 2024-08-29

**Authors:** Kenney Ki Lee Lau, Kenny Yat Hong Kwan, Jason Pui Yin Cheung, Janus Siu Him Wong, Graham Ka Hon Shea, Karlen Ka Pui Law, Kenneth Man Chee Cheung

**Affiliations:** 1 Department of Orthopaedics and Traumatology, Li Ka Shing Faculty of Medicine, The University of Hong Kong, Pokfulam, Hong Kong; 2 Department of Orthopaedics and Traumatology, The University of Hong Kong Shenzhen Hospital, Shenzhen, China

## Abstract

**Background:**

The prognosis of surgically treated subjects with early onset scoliosis (EOS) into adulthood has been lacking. We aimed to investigate the patients’ perspectives on satisfaction with surgical treatment.

**Methodology:**

We included all surgical candidates with EOS who had undergone index spinal surgery for scoliosis correction between 2009 and 2013. The minimum duration of postoperative follow-up was 10 years at the time of survey completion. Three questionnaires were used in this study, comprising the revised Scoliosis Research Society questionnaire (SRS-22r), the Patient-Reported Outcomes Measurement Information System (PROMIS-29), and the World Health Organization Quality of Life (WHOQOL-BREF). Measures of treatment satisfaction were retrieved from SRS-22r.

**Results:**

There were 29 participants who completed the survey, and thereby included in the study (i.e., a response rate of 43% and a dropout rate of 6%). Amongst them, 14, 11, and 4 individuals received posterior spinal fusion (PSF), magnetically controlled growing rods (MCGR), and traditional growing rods, respectively. The average duration after the index spinal surgery was 12.6 ± 2.2 years. Our findings revealed that self-image (across all treatment groups), sleep disruption (only in PSF), and social aspects (in both PSF and MCGR) were significantly worse when compared to the normative values. According to the multivariable linear regression model (R-square = 0.690, *p* < .001), an increase in SRS-22r mental health (*p* = .008) and PROMIS-29 social participation scores (*p* = .004) corresponded to 0.511 and 0.055 points increases in satisfaction. Every unit increase in PROMIS-29 fatigue (*p* = .043) and WHOQOL-BREF physical domain scores (*p* = .007) was in conjunction with 0.019 and 0.040 points decreases in satisfaction. SRS-22r self-image (*p* = .056) and WHOQOL-BREF environmental domain scores (*p* = .076) were included in the model but did not reach statistical significance.

**Conclusions:**

To improve the long term quality of life in surgical candidates with EOS, we demonstrated that mental health, social participation, fatigue, and physical health were significant factors associated with treatment satisfaction. Interestingly, demographic and radiographic parameters did not have a significant effect in our cohort.

## Introduction

Patients with early onset scoliosis (EOS) are diagnosed before age 10 and usually present with varying symptoms [1, 2]. While it is less common than other spinal deformities, approximately 40% of cases may progress and necessitate surgical intervention [3]. In recent years, technological advancements like the magnetically controlled growing rod (MCGR) [4], have been developed to treat this spinal condition [5]. However, there is still no consensus on the best surgical treatment option [6–9]. Due to the diverse causes and pathologies of EOS [10, 11], it remains difficult to establish a standardised protocol for use in clinical settings worldwide.

In addition to clinical justifications, the literature is full of ongoing debates concerning patient-reported outcome measures for surgical treatments in EOS [12, 13]. The MCGR is a non-invasive method for lengthening [4], and several papers have reported its advantages in terms of quality of life compared to the traditional growing rod (TGR) [14–16]. Alternatively, some studies claimed the superiority of TGR [17, 18], whilst others found no significant differences between the two methods [19]. Most importantly, there is a lack of research investigating the long term prognosis of individuals who received surgical intervention during childhood and have now reached adulthood. As a decade has passed since the implementation of MCGR, evaluating their postoperative outcomes is vital.

When it comes to treatment satisfaction, the patient’s perspective is just as crucial as radiographic success. Although several clinical parameters have been linked to quality of life following surgery [20–22], it is still uncertain whether scoliotic children are satisfied with their postoperative results. Furthermore, patient-centred data has not been thoroughly explored, as the revised 22-item Scoliosis Research Society questionnaire (SRS-22r) is the only instrument used across various age groups within the scoliosis population [23]. The efficacy of other questionnaires for participants with EOS remains unknown.

Our objective was to evaluate deformity-specific, overall health-related, and general quality of life measures in subjects with EOS and at least 10 years after their index surgery. Specifically, we compared participants who had undergone MCGR, TGR, and posterior spinal fusion (PSF). We also aimed to examine the factors perceived by patients themselves as contributing to their satisfaction with surgical treatment.

## Materials and methods

This manuscript was prepared in accordance with the strengthening the reporting of observational studies in epidemiology (STROBE) statement [24].

### Study design

The present study was a prospective cross-sectional survey. The study protocol strictly followed the Declaration of Helsinki and obtained ethical approval from the institutional review board (reference number: UW 23–303). Informed consent was attained prior to the study process being initiated.

### Setting

Our centres are the designated tertiary referral clinics for scoliosis management in Hong Kong. Potential study subjects were identified from the electronic medical health record system of the clinical data analysis and reporting system (CDARS) based on the procedure received. Eligible subjects were contacted by phone and invited to participate in the study. Those who gave verbal consent were asked to complete the self-administered questionnaires online. We utilised the REDCap platform to facilitate the construction and distribution of the survey. Data collection was implemented from August to December 2023.

### Participants

All surgical candidates with EOS who had undergone an index spinal surgery for scoliosis correction (i.e., MCGR, TGR, or PSF) between 2009 and 2013 were enrolled. These patients first presented to our clinics when they were 10 years old or below. We only included study subjects with a minimum postoperative duration of 10 years at the time when they completed the survey. Individuals were excluded if they had cognitive impairment or mental illness.

### Measurements

Three questionnaires were employed in this study, comprising SRS-22r [25], the Patient-Reported Outcomes Measurement Information System 29 profile v2.1 (PROMIS-29) [26], and the World Health Organization Quality of Life brief version (WHOQOL-BREF) [27]. The SRS-22r questionnaire is explicitly designed for scoliosis and evaluates 5 domains related to the back condition, including function, pain, appearance, mental health, and treatment satisfaction. The PROMIS-29 was developed to assess 8 different areas, namely physical function, anxiety, depression, fatigue, sleep disturbance, ability to participate in social roles and activities, pain interference, and pain intensity in individuals with chronic conditions. Additionally, the WHOQOL-BREF serves as a generic cross-cultural quality of life assessment, covering various aspects such as physical, psychological, social, and environmental domains. Demographic and radiological data were obtained from electronic medical records.

### Variables

We focused on the 2 questions in the SRS-22r questionnaire related to treatment satisfaction for further analysis (i.e., are you satisfied with the results of your back management & would you have the same management again if you had the same condition). While most questionnaires had higher scores indicating better measures, specific domains of PROMIS-29 were inversely associated with worsened health (i.e., anxiety, depression, fatigue, sleep disturbance, pain interference, and pain intensity). The scores in SRS-22r ranged from 1 to 5, whereas the scores in WHOQOL-BREF ranged from 0 to 100. These two data sets were then compared to normative values retrieved from the literature [28–33]. A variety of studies have collected data on the SRS-22r. Hasegawa et al. polled 150 respondents with an average age of 40.9 in Japan, among whom 64% were women [33]. Verma et al. carried out 2 independent investigations in the United States. One encompassed 1,019 individuals with a mean age of 14.4 and 42% females,^32^ and the other incorporated 450 participants with an average age of 16.0 and 62% females.^30^ Daubs et al. performed another research in the United States involving a larger cohort of 3,052 people with an average age of 14.6, where 51% were women [31]. Regarding the WHOQOL-BREF, Cruz et al. probed 751 Brazilians with an average age of 41.0 with 62% being women [29], and Noerholm et al. took a survey of 1,101 Danes with undisclosed average age with 52% of them being women [28]. Furthermore, the PROMIS-29 offered a unique metric by converting raw data into T-scores (with a mean of 50 and a standard deviation of 10). Its affiliated organisation defined the cut-off values with reference to the United States population. Regarding the demographics, we documented ages at presentation, surgery, and survey, gender, anthropometry, diagnosis, postoperative complications, and final fusion for subjects who underwent growing rod procedures. Moreover, we measured the preoperative and most recent Cobb angles of the major curve.

### Statistical analysis

In light of the primary objective, we performed a Quade non-parametric analysis of covariance to compare among groups, adjusting for confounders when a significant between-group difference was observed in demographic and radiological parameters. A Chi-square test was also conducted for categorical data. For comparisons with normative values, we used a one-sample T-test to evaluate each outcome measure. Moreover, we employed a backward stepwise multivariable linear regression model to identify factors associated with surgical treatment satisfaction, using Pearson’s correlation coefficient to determine which factors to input into the model. The analyses were performed exploiting the SPSS Statistics version 29.0, with a significance level set at *p* < .05.

## Results

**Fig. 1:**
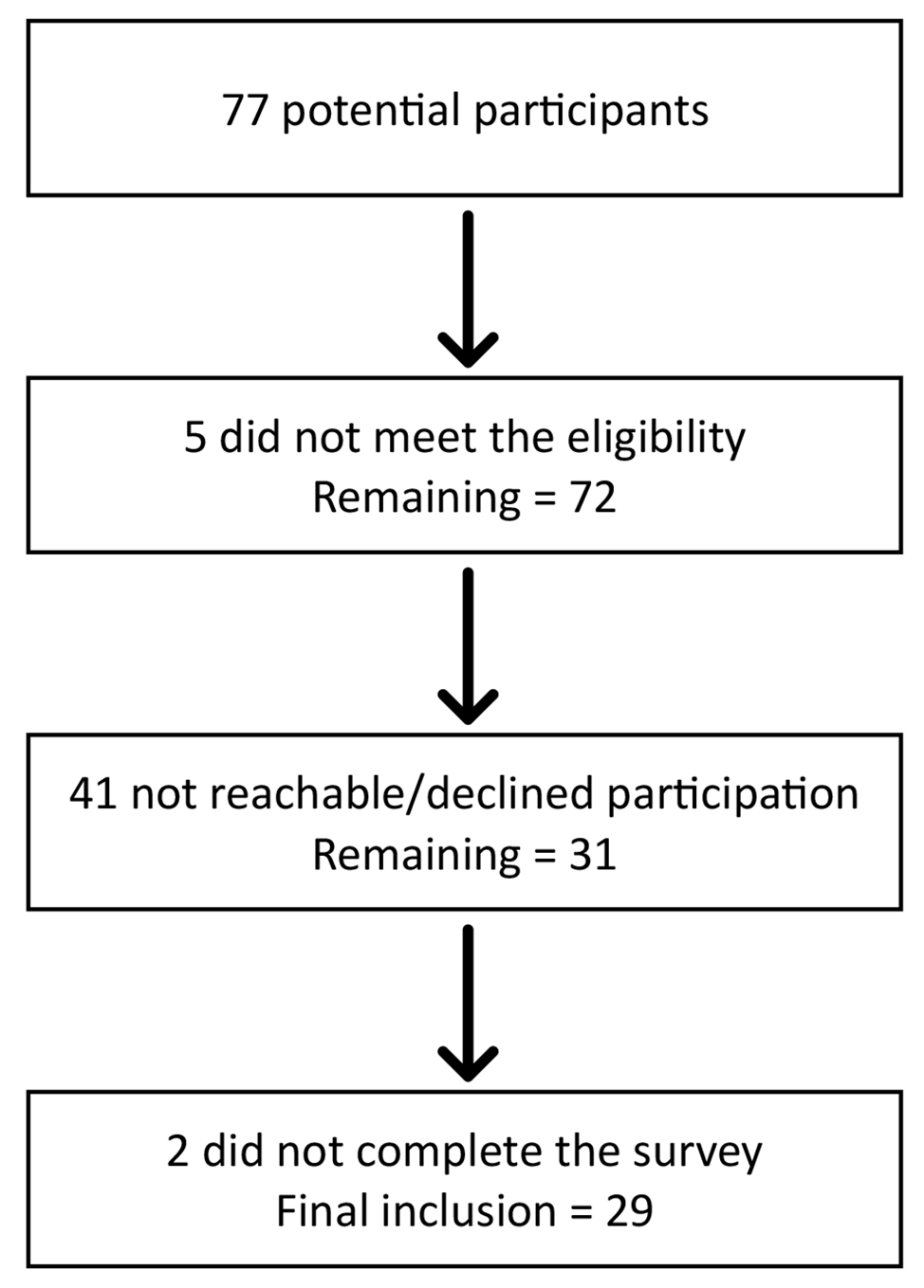
Flow diagram of participant selection

During the study period, 77 subjects with EOS underwent scoliosis correction (Fig. 1). Five of them were excluded due to a history of psychiatric disorders. Out of the remaining candidates, 26 could not be reached, and 15 declined participation. Of the 31 who consented to participate, 29 completed the survey and were included in the present study (i.e., a response rate of 43% and a dropout rate of 6%). Among the participants, 48% received PSF, 38% received MCGR, and 14% received TGR. On average, 12.6 ± 2.2 years had passed since their index spinal surgery. As expected, there were few differences in characteristics among the groups, including the age and the complication rate. Older patients are more likely to undergo definitive fusion, while growing rods are more prone to complications compared to fusion. These two factors were adjusted in subsequent analyses. Participants’ information can be found in Table 1.

**Table 1:** Characteristics of participants

	Overall EOS	PSF	MCGR	TGR	Significance
Sample size	29	14	11	4	/
Females	69%	71%	64%	75%	0.881
Age	Presentation: 7 ± 4 Operation: 11 ± 3 Survey: 24 ± 3	Presentation: 8 ± 3 Operation: 13 ± 2 Survey: 25 ± 2	Presentation: 7 ± 4 Operation: 9 ± 3 Survey: 21 ± 4	Presentation: 7 ± 6 Operation: 9 ± 4 Survey: 25 ± 2	0.876 0.003* 0.008*
Anthropometry	Height: 155 ± 9 cm Weight: 48 ± 12 kg BMI: 20.1 ± 5.3	Height: 154 ± 9 cm Weight: 49 ± 11 kg BMI: 20.5 ± 3.8	Height: 156 ± 12 cm Weight: 49 ± 14 kg BMI: 20.5 ± 7.3	Height: 154 ± 4 cm Weight: 43 ± 7 kg BMI: 18.0 ± 3.9	0.580 0.501 0.435
Diagnosis	52% idiopathic 17% congenital 10% neuromuscular 21% syndromic	57% idiopathic 14% congenital 14% neuromuscular 14% syndromic	46% idiopathic 18% congenital 0% neuromuscular 36% syndromic	50% idiopathic 25% congenital 25% neuromuscular 0% syndromic	0.547
Treatment	48% PSF 38% MCGR 14% TGR	/	Final fusion: 73%	Final fusion: 75%	0.930
Complication rate	38%	14%	55%	75%	0.031*
Pre-op Cobb	59° ± 17°	62° ± 16°	55° ± 19°	62° ± 18°	0.477
Latest Cobb	26° ± 14°	23° ± 12°	29° ± 17°	29° ± 13°	0.580

Note. EOS = early onset scoliosis; PSF = posterior spinal fusion; MCGR = magnetically controlled growing rod; TGR = traditional growing rod; BMI = body mass index; * as significant

**Fig. 2:**
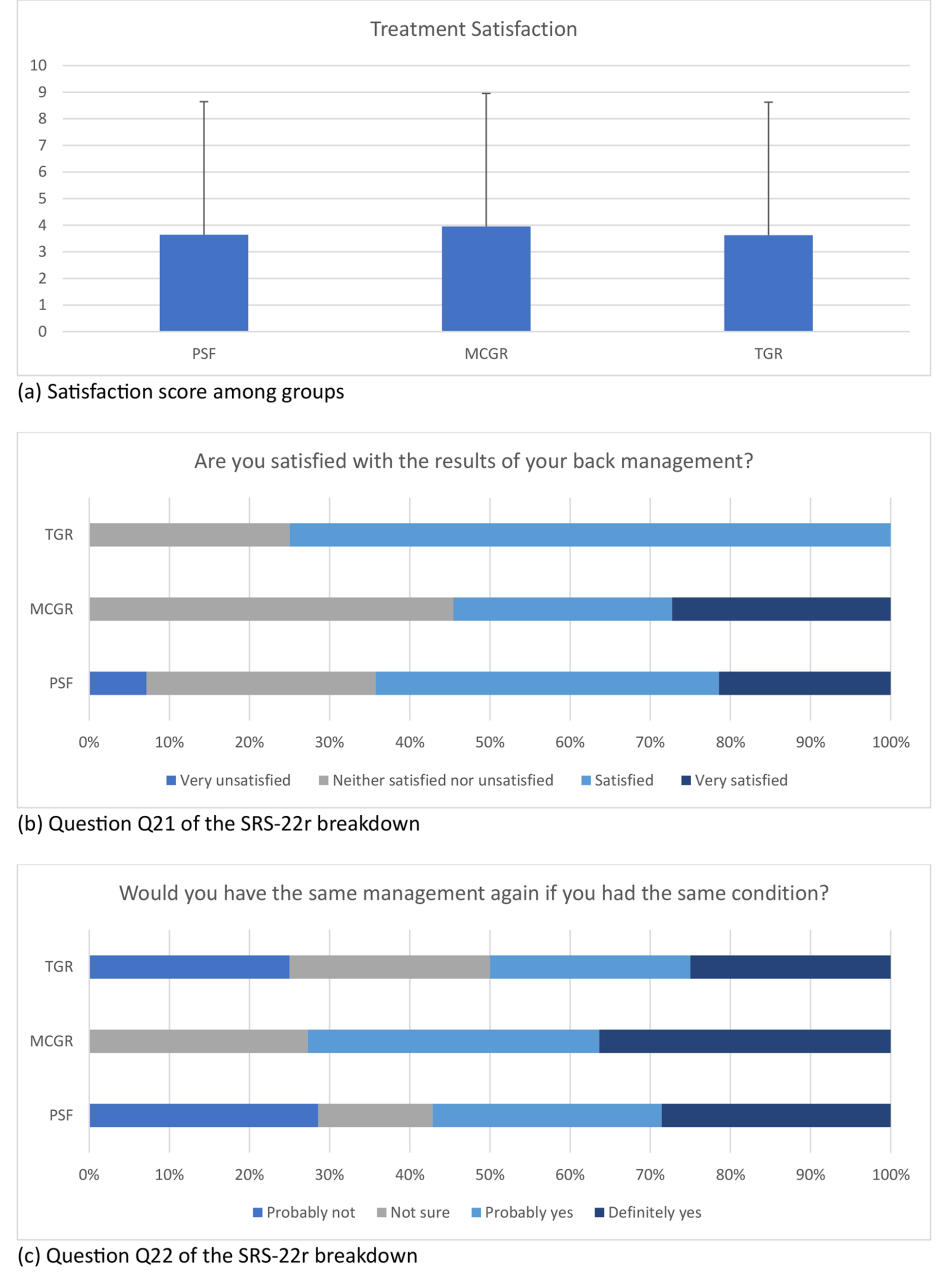
Level of satisfaction

The satisfaction with treatment, as assessed by SRS-22r, showed no significant differences among groups (Fig. 2). The level of satisfaction with back management was also similar across each group. Additionally, there was no statistical significance in participants’ willingness to undergo the same treatment again. Concerning other quality of life measures, MCGR subjects showed significant differences only in lower function (*p* < .041) and social participation scores (*p* < .045) than those with TGR.

**Fig. 3:**
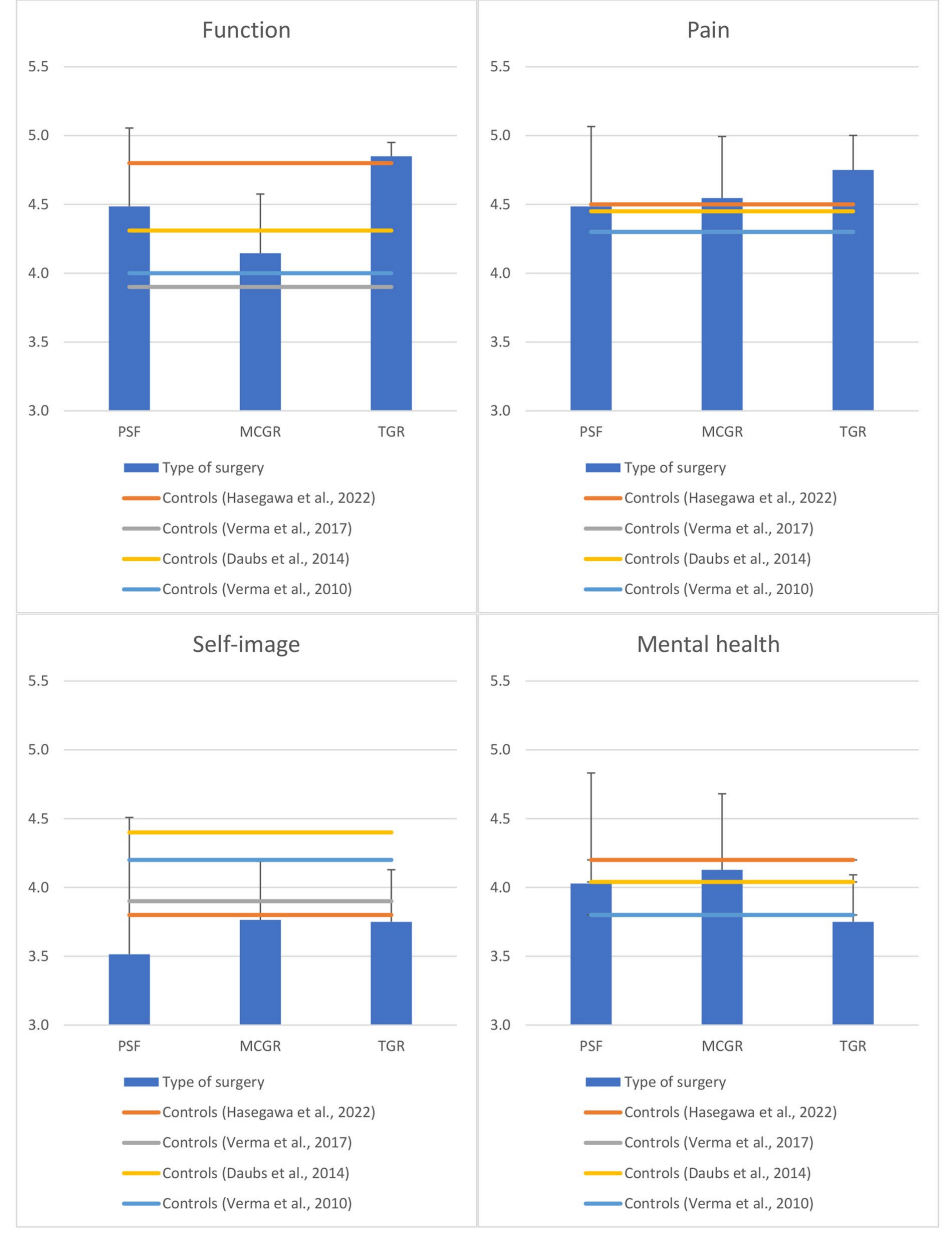
SRS-22r scores

**Fig. 4:**
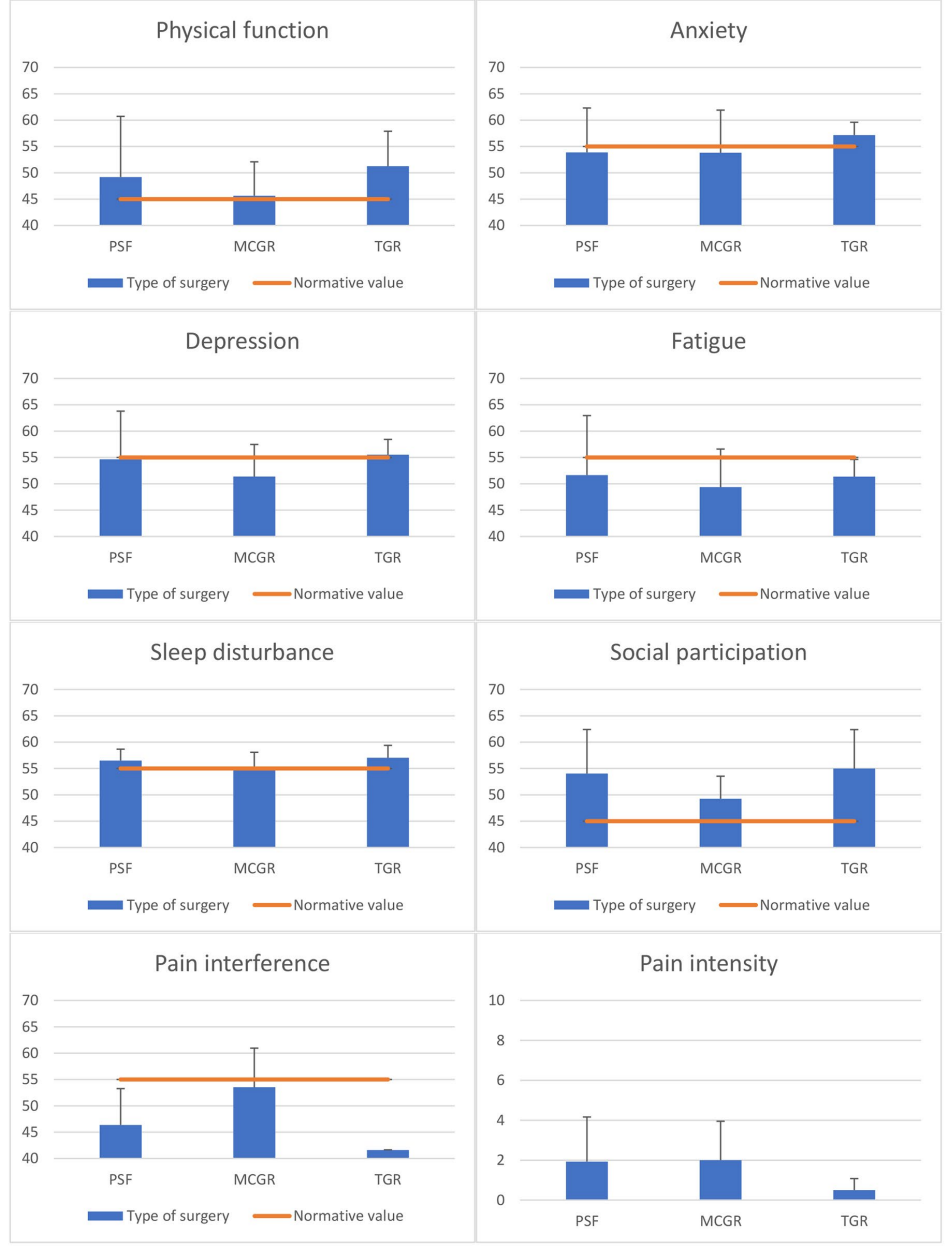
PROMIS-29 scores

**Fig. 5:**
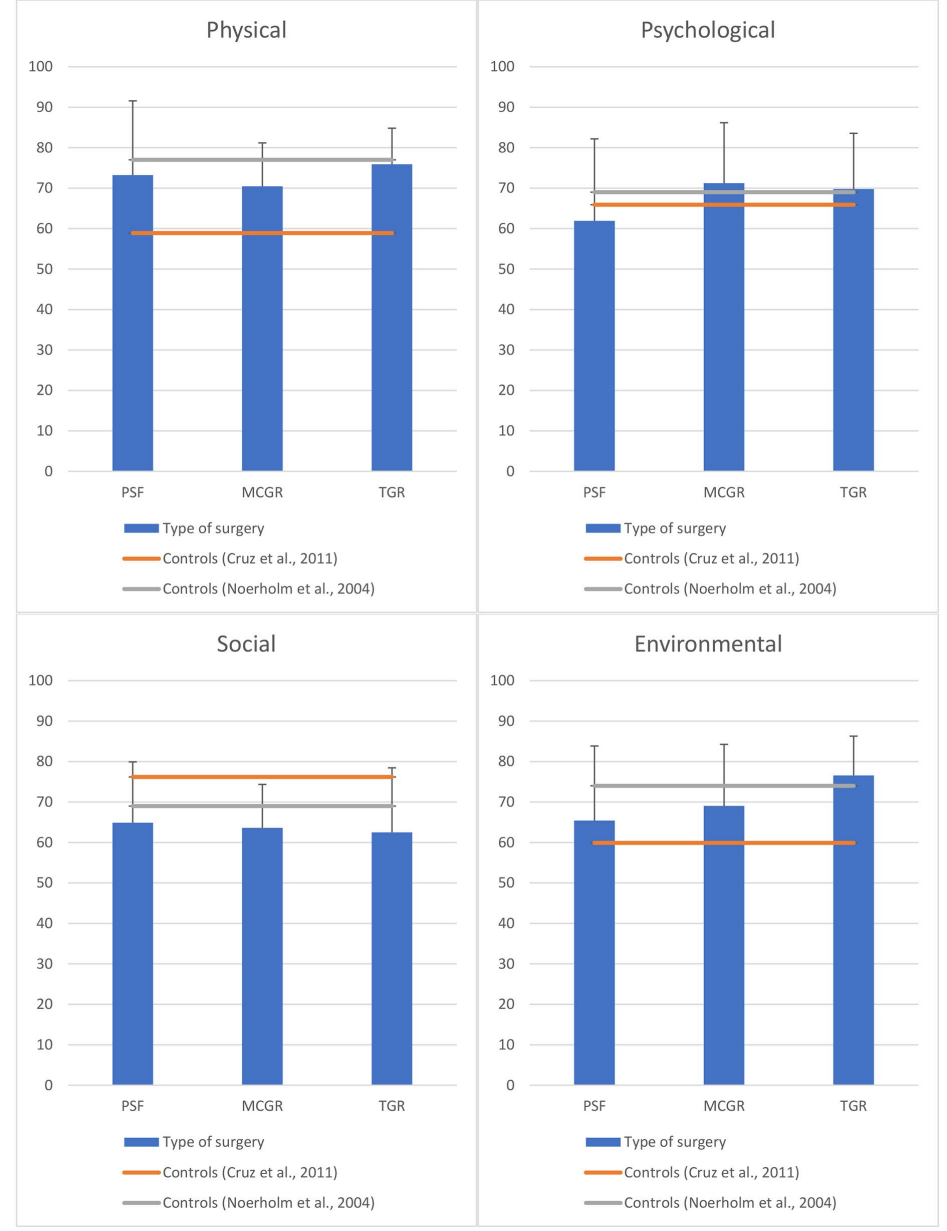
WHOQOL-BREF scores

In SRS-22r, all groups exhibited improved function and poorer self-image compared to the norms and TGR showed better pain management (Fig. 3). PROMIS-29 revealed less fatigue in the MCGR group, increased sleep disturbance in the PSF group, reduced social participation in both PSF and MCGR groups, and decreased pain interference in both PSF and TGR groups (Fig. 4). Furthermore, all study participants demonstrated higher physical capability compared to the normative values of WHOQOL-BREF (Fig. 5). PSF and MCGR subjects had lower social scores, while TGR subjects had better environmental scores relative to the healthy population.

The following regression analyses encompassed all 29 participants. The univariate correlation analyses revealed that SRS-22r function (*r* = .438, *p* = .017), SRS-22r pain (*r* = .560, *p* = .002), SRS-22r self-image (*r* = .692, *p* < .001), SRS-22r mental health (*r* = .605, *p* < .001), PROMIS-29 fatigue (*r*=-.545, *p* = .002), PROMIS-29 social participation (*r* = .457, *p* = .013), PROMIS-29 pain intensity (*r*=-.554, *p* = .002), WHOQOL-BREF physical domain (*r* = .463, *p* = .011), WHOQOL-BREF psychological domain (*r* = .439, *p* = .017), and WHOQOL-BREF environmental domain (*r* = .574, *p* = .001) were associated factors for treatment satisfaction. However, demographic and radiological parameters were not found to be significantly related. All these associated factors were used for further analysis.

The multivariable regression model with a value of R-square at 0.690 indicated that an increase in SRS-22r mental health (*p* = .008) and PROMIS-29 social participation scores (*p* = .004) were associated with 0.511 and 0.055 point increases in satisfaction, respectively. Conversely, every unit increase in PROMIS-29 fatigue (*p* = .043) and WHOQOL-BREF physical domain scores (*p* = .007) corresponded to 0.019 and 0.040 point decreases in satisfaction, respectively. While SRS-22r self-image (*p* = .056) and WHOQOL-BREF environmental domain scores (*p* = .076) were included in the model, they did not achieve statistical significance.

## Discussion

Our study examined the quality of life in adults with EOS who underwent surgical treatment after a minimum of 10 years from their index surgery. We found that treatment satisfaction and other domains were generally similar among PSF, MCGR, and TGR groups. However, various measures remained below the levels of the healthy population. To enhance the long term quality of life for surgical candidates, we identified mental health, social participation, fatigue, and physical health as significant factors associated with treatment satisfaction.

The decision to undergo surgical intervention for EOS, as well as the surgical treatment strategy, remains a controversial issue [34–36]. While achieving optimal clinical outcomes is crucial, patient-based outcomes are equally important. Previous literature supporting MCGR has been using the Early Onset Scoliosis Questionnaire for evaluation [14–16], a caregiver-based measurement [37], and reported its superiority over TGR in terms of satisfaction [14], financial burden [14], transfer [15], fatigue [15], and pulmonary function [16]. However, conflicting results have been found using other measurements [17, 18]. While the evidence was based on children with EOS, our findings showed no differences among surgical treatment options when patients reached adulthood. Although the choice of quality of life measurement may influence the results [38], the ideal treatment for EOS is yet to be determined.

Even after more than 10 years post-operation, a portion of the study subjects still experienced mental distress compared to healthy controls. Our data demonstrated that self-image (all treatment groups), sleep disturbance (PSF only), and social domain (PSF and MCGR) were significantly impacted among participants. The negative appearance of individuals with scoliosis is well-recognised in academic and clinical settings [39]. In theory, surgical intervention corrects spinal curvature, thereby improving rib and loin humps [40]. Nevertheless, this may be limited by severe curves at the time of surgery [41, 42]. The issue is that no early detection method is available for scoliosis at an early age [43], so patients may already present with severe curves at their initial appointment [44]. Moreover, sleep problems and social relationships have been inadequately described in EOS [20, 45], and their causal relationship with scoliosis development remains unknown.

Patient satisfaction is a critical indicator of treatment success in EOS. Surprisingly, there is a lack of studies examining this measure, and the corresponding associated factors are poorly understood. Despite some inherent elements, such as the deformity itself [6], or its aetiology [10–12], may be affecting the patient’s psychological health, modifiable physical factors, such as spinal height [21], are still available for additional improvement. These separately identified risk and prognostic factors have not been validated for their combined effects on quality of life measures in EOS, and psychological measures have not been considered until this study. Our analysis included one physical factor and three psychological factors in the final regression model for treatment satisfaction, suggesting the potential implication of mental health components.

The current findings recognised the presence of gaps in knowledge. Specifically, there is a lack of long-term studies assessing the radiological and psychological characteristics of individuals with EOS. It remains unclear how their physical and mental well-being will develop in later adulthood and the impact of various treatments and risk factors on their outcomes. Additionally, there is a strong desire to understand their quality of life compared to a healthy population. It is uncertain whether treatments for EOS effectively enable individuals to lead a normal life similar to those without scoliosis. Furthermore, there is a lack of adequate research investigating the effectiveness of psychological interventions in improving treatment success despite the importance of psychosocial factors being aroused.

The limitations of this study involve a small sample size and low response rate. While the study lacked concurrent control groups, we attempted to compare results with historical cohorts. The cross-sectional design restricts the ability to establish causal relationships. Additionally, the study relies on self-reported measures, which may lead to recall bias. Our comparison did not include alternative surgical interventions, such as the Shilla growth guidance technique and vertically expanding prosthetic titanium rib. Finally, we did not examine the long term effects of different treatments and risk factors on the physical and mental health outcomes of individuals with EOS.

## Conclusions

Our study found that treatment satisfaction and other quality of life domains were generally similar among PSF, MCGR, and TGR groups in adults with EOS who underwent surgical treatment for over 10 years. However, various measures remained below the average of the general population. Mental health, social participation, fatigue, and physical health were significant factors associated with treatment satisfaction. The decision to undergo surgical intervention for EOS remains a controversial issue, and the ideal treatment for EOS is yet to be determined. The current results highlighted the need for long term research assessing the radiological and psychological characteristics of individuals with EOS and the effectiveness of psychological interventions in improving treatment success.

## Data Availability

No datasets were generated or analysed during the current study.
